# *MiR-375* attenuates injury of cerebral ischemia/reperfusion via targetting Ctgf

**DOI:** 10.1042/BSR20171242

**Published:** 2017-12-22

**Authors:** Jianying Ou, Li Kou, Lingyan Liang, Chaogang Tang

**Affiliations:** 1 Department of Rehabilitation Medicine, The Fifth Affiliated Hospital of Sun Yat-Sen University, Zhuhai 519000, P.R. China; 2 Department of Neurology, The Fifth Affiliated Hospital of Sun Yat-Sen University, Zhuhai 519000, P.R. China

**Keywords:** Connective tissue growth factor, Ischemia/Reperfusion, MiR-375

## Abstract

Ischemic stroke is the leading cause of disability and deaths worldwide. MiRNAs have been shown to play an important role in development and pathogenesis of the nervous system. However, the precise function and mechanism of miRNAs are not fully understood in the brain injury induced by ischemia/reperfusion (I/R). Herein, our study showed that *miR-375* expression was significantly down-regulated in the rat I/R brain. With the *in vivo* and *in vitro* I/R stroke models, we found that *miR-375* mimic provides significant protection from injury to cerebral I/R, which is reflected by reduced infarct volumes and cell apoptosis, and increased proliferation and migration of PC12 cells. Mechanistically, our findings showed that *miR-375* binds to 3′-UTR region of *Ctgf* mRNA, subsequently leading to the decreased expression of Ctgf in the I/R brain. Furthermore, we showed that *miR-375*/Ctgf-mediated protective effects are associated with p21/PI3K/Akt signaling pathways. Our findings thus provide a new insight into the mechanism of cerebral I/R injury and pave a potential new way for the therapy of cerebral I/R injury.

## Introduction

Ischemic stroke is the main cause of permanent injury, disability, and deaths in the world. Ischemic stroke is usually treated by intravenous thrombolytic with alteplase or intra-arterial mechanical thrombectomy with stent retriever devices. However, this therapy, also known as cerebral ischemia/reperfusion (I/R), can lead to increased brain injury. Accumulating evidence shows that apoptosis and death of neural cells after I/R are the leading causes of aggravated brain injury [1,2]. Thus, it should be an important strategy to perform anti-apoptotic treatment as early as possible following cerebral I/R. However, the exact mechanisms underlying cerebral I/R-induced neuronal death and dysfunction are not fully understood.

MiRNAs are a class of small noncoding RNA molecules (~22 nts) found in most eukaryotes, including humans [3]. Its discovery has largely broadened our understanding of the mechanisms that regulate gene expression in various physiological and pathological conditions. It is known that miRNAs can induce translational silencing and repress target protein production through binding to the 3′-UTR region of the target mRNA.

In neural system, a large number of studies has suggested that miRNA plays an important role in the development and pathogenesis of the nervous system [4–6]. For example, *miR-145* has recently been shown to regulate neural stem cell differentiation through the Sox2-Lin28/let-7 signaling pathway [7]. *MiR-455-3p* has been proven to be a potential peripheral biomarker for Alzheimer’s disease [8]. In the ischemic brain, few studies evaluated miRNA profiling and changes. Amongst these, our interest was aroused by *miR-375* which is specifically expressed in the pancreatic islets, brain, and spinal cord. Wang et al. first showed that *miR-375* expression was down-regulated in the cerebral I/R rat [9]. However, they did not provide direct evidence that *miR-375* expression is related to injury of I/R. Recently, Bhinge et al. [10] showed that *miR-375* was essential for motor neurone development and degeneration. Therefore, we hypothesized that down-regulation of *miR-375* might be an important contributor to increased injury induced by I/R.

In the present study, a new target Ctgf of *miR-375* was identified using bioinformatics software. With middle cerebral artery occlusion (MCAO) rat model and *in vitro* hypoxia/reoxygenation (H/R) PC12 model, we provided direct evidence that *miR-375* shows a protection from injury of cerebral I/R through targetting *Ctgf* mRNA and decreasing Ctgf protein expression. Our findings provide a new insight into the mechanism of cerebral I/R injury and pave a potential new way for the therapy of cerebral I/R injury.

## Materials and methods

### Luciferase assay

The human Ctgf 3′-UTR DNA sequence that was predicted to interact with *miR-375* and the mutant Ctgf 3′-UTR DNA sequence were amplified and inserted into psi-CHECK2 *Renilla*/firefly Dual-luciferase expression vector (Promega, Mullion, WI, U.S.A.), with the following primers: Ctgf 3′-UTR-WT: 5′-CCGCTCGAGAAACTGATAGCCTCAAACTCC-3′ (forward) and 5′-ATTTGCGGCCGCTAAACTGCCTCCCAAACC-3′ (reverse). Ctgf 3′-UTR-MUT: 5′-TAAAATCACTGTTGGATCTGTCATGGCCTTTATTAAG-3′ (forward) and 5′-CTTAATAAAGGCCATGACAGATCCAACAGTGATTTTA-3′ (reverse). *MiR-375* mimic, a chemically synthesized exogenous *miR-375* mature sequence mimic and scramble negative control (NC) miRNA were supplied by Guangzhou RiboBio, China. HEK-293T cells were co-transfected with the reporter constructs and *miR-375* mimic or NC with Lipofectamine 2000 (Invitrogen, Carlsbad, California, U.S.A.). Luciferase activities were determined after 48 h using the Dual-luciferase reporter assay system (Promega) on Enspire (PerkinElmer, Waltham, MA, U.S.A.). Data are presented calculated as the ratio of *Renilla* luciferase activity in cell lysates to firefly luciferase.

### I/R rat brain model establishment and drug treatment

Male adult Sprague–Dawley rats (250–280 g) were obtained from Animal Center of Southern Medical University (Guangzhou, China). All procedures conducted with rats were in accordance with standard procedures approved by the Institutional Ethical Committee. Rats were anesthetized by 10% chloral hydrate (350 mg/kg) and subjected to MCAO. Briefly, after vessel isolation, a 3-0 monofilament nylon suture (Johnson and Johnson, Somerville, NJ, U.S.A.) was inserted into the internal carotid artery and anterior cerebral artery through the external carotid artery to occlude the MCA. After 2 h of operation, the suture was removed to induce reperfusion, and the rats were kept in intensive care incubator under 37°C for 24 h.

Rats were randomly divided into five groups: (i) Sham: threading without occluding, persisting perfusion (*n*=5); (ii) I/R group: 2 h ischemia and 24 h reperfusion (*n*=5); (iii) I/R + NC: rats were administrated with scramble miRNA (NC; 20 μΜ/l, intracerebroventricular injection) after ischemia treatment (*n*=5). (iv) I/R + mimic: rats were administrated with mimic (20 μΜ/l, intracerebroventricular injection) after ischemic treatment (*n*=5). (v) I/R + FNS: fastigial nucleus electrostimulation (FNS) of rats was electrically prestimulated for 1 h, and rats were treated in the same way as I/R group 24 h later (*n*=5). *MiR-375* mimic and NC were resolved in artificial cerebrospinal fluid (Harvard Apparatus, 59-7316, U.S.A.), while Sham, I/R group, and I/R + FNS were intracerebroventricularly injected with artificial cerebrospinal fluid.

### Measurement of infarct volume

After 24-h reperfusion, rats were decapitated, then the brains were rapidly isolated and cut into 2-mm thick coronal sections. The brain sections were stained with 2% 2,3,5-Triphenyltetrazolium Chloride (TTC; Sigma, 17779, U.S.A.) at 37°C for 30 min followed by immersion in 10% formalin overnight.

### Histological assessment

After I/R treatment, some rat brains were fixed in 10% neutral buffered formalin (NBF) and embedded in paraffin. Paraffin sections were stained with Hematoxylin and Eosin (H&E) or terminal deoxynucleotidyl transferase mediated dUTP nick-end labeling (TUNEL; Roche, 11966006001, Germany) or antibody against Ctgf (Santa Cruz Biotechnology, sc-101586, U.S.A.). The images of H&E and immunohistochemistry were measured through phase-contrast light microscope (Leica, Wetzlar, Germany), while the images of TUNEL were examined under the inverted fluorescence microscope (Leica). For TUNEL assay, the rate of apoptotic events in total cells was calculated.

### Measurement of intracellular reactive oxygen species, malondialdehyde, and SOD

We employed intracellular reactive oxygen species (ROS; j&l Biological, JL13542, Shanghai, China), malondialdehyde (MDA; j&l Biological, JL13297, Shanghai, China) and superoxide dismutase (SOD; j&l Biological, JL11065, Shanghai, China) measurement to indicate the protective role of *miR-375* mimic in inflammation caused by I/R. The rat brain tissue samples were homogenized for 10 min, and the intracellular ROS was determined using 2′,7′-dichlorofluorescin diacetate (DCFH-DA, Invitrogen). After centrifugation at 4000 rpm for another 10 min, MDA and SOD activities in the supernatant of brain tissue lysates were measured according to its provider’s instructions.

### H/R PC12 cell model establishment

P12 cell culture medium was replaced by ischemic buffer (137 mM NaCl, 0.49 mM MgCl, 12 mM KCl, 0.9 mM CaCl_2_.H_2_O, 4 mM HEPES, 10 mM deoxyglucose, and 20 mM sodium lactate (pH 6.2)), and cells were transferred to an anoxic chamber containing 1% O_2_, 94% N_2_, and 5% CO_2_ at 37°C to simulate hypoxia. Cells were subjected to simulated hypoxia injury for 6 h, after which the hypoxia buffer was replaced with DMEM containing 1% FBS and cultured in a normoxic incubator containing 95% O_2_ and 5% CO_2_ at 37°C (simulated reperfusion) for 48 h.

### Real-time PCR

Total RNA from rat brains and cells was extracted with RNAprep pure tissue kit (Tiangen Biotech Co., Ltd., Beijing, China) and RNeasy Mini Kit (Qiagen, Germantown, MD, U.S.A.), respectively. The expression levels of *miR-375* and Ctgf were amplified by quantitative real-time RT-PCR. It was carried out using SuperScriptIII Platinum SYBR Green One-Step qRT-PCR kit (Invitrogen, Carlsbad, California, U.S.A.) by an ABI PRISM 7500 Fast Real-time PCR instrument (Applied Biosystems, Foster City, CA, U.S.A.). It was carried out with the following procedures: 94°C for 2 min, 94°C for 20 s, followed by 40 cycles of 58°C for 20 s and 72°C for 20 s. The specific primers used in quantitative real-time RT-PCR were displayed as follows: *miR-375*: forward, 5′-ACACTCCAGCTGGGTTTGTTCGTTCGGCTCGC-3′; reverse, 5′-CTCAACTGGTGTCGTGGAGTCGGCAATTCAGTTGAGTCACGCGA-3′; Ctgf: forward: 5′-CTTCTGCGATTTCGGCTCC-3′; reverse, 5′-TACACCGACCCACCGAAGA-3′; GAPDH: forward, 5′-GGTGGTCTCCTCTGACTTCAACA-3′; reverse, 5′-GTTGCTGTAGCCAAATTCGTTGT-3′; U6: forward, 5′-CTCGCTTCGGCAGCACA-3′; reverse, 5′- AACGCTTCACGAATTTGCGT-3′. The data were analyzed according to the 2^−ΔΔ*C*^_t_ method.

### Western blot assay

For whole-cell extract preparation, the brain tissue and cells were lysed in tissue protein extraction reagent (T-PER) and mammalian protein extraction reagent (M-PER) containing halt protease inhibitor cocktail (Thermo Fisher Scientific, Waltham, MA, U.S.A.), respectively. Equal amount of proteins was subjected to SDS/PAGE and then transferred on to PVDF membrane (Millipore, Billerica, MA, U.S.A.). After being blocked by 5% milk, the membranes were incubated with antibodies against Ctgf, PI3K, AKT, p-AKT, p21, and GAPDH (Cell Signaling Technology, Beverly, MA, U.S.A.). Last, corresponding secondary HRP conjugated antibodies were applied and the signals of targetted proteins were visualized using the ECL chemiluminescent detection kit (GE Healthcare Life Sciences, Pittsburgh, PA, U.S.A.).

### Cell counting kit-8 assay

Cell viability was measured using Cell Counting Kit-8 (CCK-8, Dojindo, Kumamoto, Japan). H/R PC12 cells were treated with scramble NC miRNA or *miR-375* mimic for 24, 48, and 72 h. After washing with PBS three times, a 1:10 diluted CCK-8 solution was added to the medium and incubated at 37°C for 1 h. The absorbance was measured by microplate reader at 450 nm.

### Hoechst staining assay

H/R PC12 cells treated with NC or *miR-375* mimic were cultured in six-well plates, and Hoechst 33258 (Sigma–Aldrich, St. Louis, MO, U.S.A.) was added to the culture medium and incubated for 10 min, cells were then washed with PBS three times, and the counts of Hoechst-positive nuclei were detected by inverted fluorescence microscope using a 365-nm filter for Hoechst 33258.

### Immunofluorescence staining

Immunofluorescence staining was performed to evaluate the expression of Ctgf. H/R PC12 cells treated with NC or *miR-375* mimic were fixed with 4% paraformaldehyde and permeabilized in 0.5% Triton X-100. After being blocked with 3% BSA, cells were incubated with primary antibodies against Ctgf and DAPI. Next, cells were stained with the FITC-conjugated and Alexa Fluor 488-conjugated secondary antibodies (BD Biosciences, San Jose, CA, U.S.A.). Finally, images were taken under the inverted fluorescence microscope.

### Transwell assay

Cell invasion was determined by the transwell assay. After transfection for 48 h, PC12 cells in serum-free medium were transferred into the upper Matrigel-coated invasion chambers (BD Biosciences) and medium containing 10% FBS was added to the lower chamber. After 24 h of incubation at 37°C with 5% CO_2_, the invading cells through the membrane were fixed with 95% ethanol, stained with 0.1% Crystal Violet, and imaged under the microscope. The percentage of migrated cells was quantitated by counting five independent visual fields.

### EdU imaging assay

For EdU staining assay, cells were permeabilized and stained with Click-iT EdU Imaging Kit (Invitrogen, Carlsbad, CA, U.S.A.) according to manufacturer’s instructions. Briefly, the H/R cells were incubated with *miR-375* mimic and pcDNA3.0-Ctgf or pcDNA3.0-vector for 24 h. After removing the culture medium, the cells were fixed with 1 ml of 3.7% formaldehyde in PBS for 15 min and incubated with 0.5% Triton X-100 in PBS for 20 min. Then, the cells were incubated with 0.5 ml of Click-iT reaction cocktail at room temperature for 30 min in the dark. After removal of the reaction cocktail, cells were washed with 3% BSA in PBS followed by PBS. After addition of DAPI in PBS, cells were incubated for 20 min at room temperature in the dark. The cells were then washed with PBS, and images were obtained under the inverted fluorescence microscope.

### Statistical analyses

Statistical analyses were performed using GraphPad Prism 6.0 software. Data were expressed as mean ± S.D., and analyzed by one-way ANOVA with multiple comparisons test (three or more datasets in a group). Differences were considered to be statistically significant when *P*<0.05.

## Results

### *MiR-375* targetting 3′-UTR region of *Ctgf* mRNA

**Figure 1:**
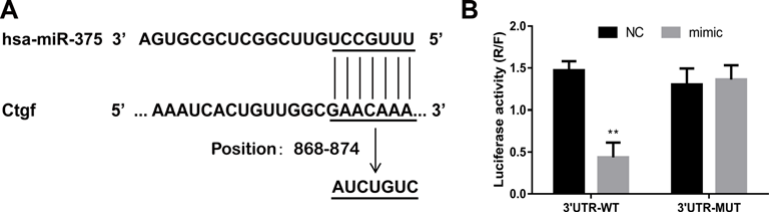
*MiR-375* targets 3′-UTR region of *Ctgf* mRNA (**A**) Bioinformatics analysis shows that *miR-375* binds to 3′-UTR region (position 868–874) of Ctgf through base-complementary pairing. The mutant site in 3′-UTR region of *Ctgf* mRNA was also shown. (**B**) HEK-293T cells were co-transfected with psi-CHECK2-Ctgf-3′-UTR (WT or MUT) and *miR-375* mimic (or NC). After 48 h, luciferase activity was calculated as the ratio of *Renilla* luciferase activity to firefly luciferase. The Dual-luciferase assay showed that Ctgf was the downstream target of *miR-375*. The experiments were performed in triplicate and each value represented mean ± S.D. **P<0.01.

To determine the target gene of *miR-375*, we used bioinformatics software of MirTarget algorithm and miRDB to predict downstream target gene of *miR-375*. The data showed that *miR-375* could bind to 3′-UTR region of *Ctgf* gene (Figure 1A), which has been associated with neural injury and death [11,12]. Next, a Dual-luciferase reporter assay was performed to confirm that *Ctgf* mRNA is *bona fide* target of *miR-375*. The result demonstrated that the relative luciferase activity of the wild-type Ctgf 3′-UTR reporter was significantly suppressed in the HEK-293T cells with the treatment of *miR-375* compared with the cells treated with miR-NC. In contrast, the luciferase activity of the binding site mutant Ctgf 3′-UTR reporter was unaffected by co-transfection of *miR-375* mimic compared with NC co-transfected (Figure 1B). These results suggest that *miR-375* might provide the protection in the injury of cerebral I/R through inhibiting Ctgf expression.

### *MiR-375* mimic reduces infarction volumes in I/R rat brain

**Figure 2:**
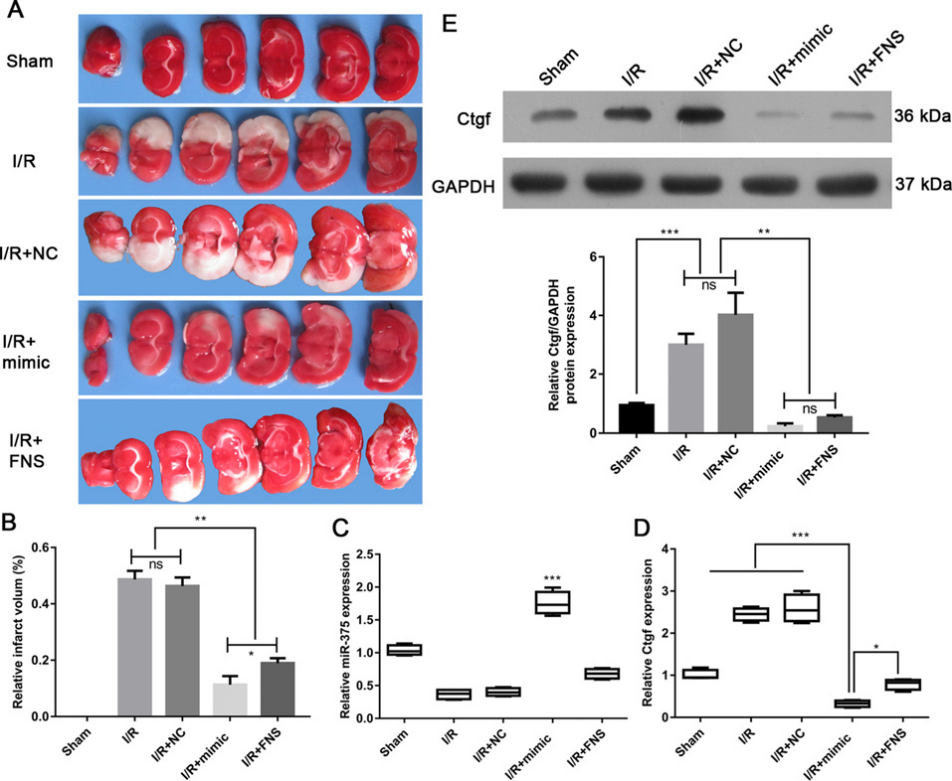
.*MiR-375* attenuated infarction volumes in I/R rat brain (**A**) TTC staining of representative coronal sections after I/R. The relative infarct area percentage was evaluated by observing the unstained infarcted tissue zone (white) and the stained normal tissue zone (red). (**B**) The protein expression of Ctgf in Sham rat brains and I/R rat brains treated with NC, mimic, and FNS was analyzed by Western blotting. (**C**,**D**) RNA expression of *miR-375* (C) and Ctgf (D) in Sham rat brains and I/R rat brains treated with NC, mimic, and FNS was analyzed by qRT-PCR. (**E**) The protein expression of Ctgf in sham rat brains and I/R rat brains treated with NC, mimics and FNS was analyzed by western blotting. The experiments were performed in triplicate and each value represented mean ± S.D. ***P<0.001 compared with I/R and I/R + NC, *P<0.05 compared with I/R + mimic. **p<0.01 compared with I/R+mimic or I/R+FNS. NS: not significance.

To demonstrate the protective role of *miR-375* against brain I/R injury, we first established the model of I/R rat brain and determined the effect of *miR-375* mimic on infarction volumes by TTC staining, with Sham, I/R, I/R + NC, I/R + FNS as control. As shown in Figure 2A, the infarct region was observed in the brain of both I/R and I/R + NC groups. However, the infarct volume was significantly reduced in the I/R group treated with *miR-375* mimic or FNS (Figure 2B). In the mean time, we detected the expression of Ctgf associated with the injury of cerebral I/R. Our results showed that expression of Ctgf mRNA and protein were significantly decreased in the I/R group treated with *miR-375* mimic and FNS compared with the I/R group with NC treatment or without treatment (Figure 2D and 2E). In line with this, the down-regulation of Ctgf in the *miR-375*-treated I/R group was linked to the up-regulation of *miR-375* (Figure 2C). Of note, FNS-mediated down-regulation of Ctgf was not linked to *miR-375* (Figure 2C,D). This result suggested that *miR-375* does protect rat from injury of cerebral I/R.

### *MiR-375* suppresses apoptosis and oxidative stress caused by I/R injury

**Figure 3:**
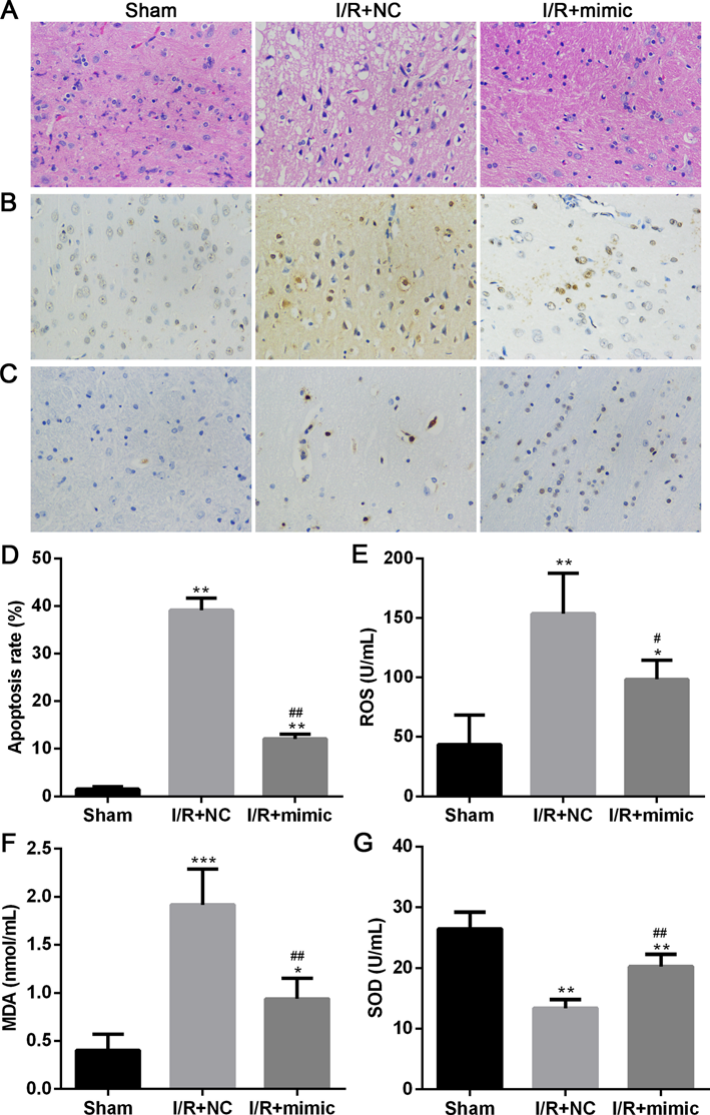
The effect of *miR-375* on apoptosis and oxidative stress in I/R rat brain (**A**–**C**) Histological assessment in I/R rat brains. The H&E staining (A), TUNEL staining, (B) and immunohistochemistry staining (C) in Sham group and I/R groups treated with NC or *miR-375* mimic. (**D**) The apoptosis rate was calculated according to the TUNEL staining assay. (**E**–**G**) Measurement of ROS (E), MDA (F) and SOD (G) levels in Sham group and I/R groups treated with NC or *miR-375* mimic. The experiments were performed in triplicate and each value represents mean ± S.D. *P<0.05, **P<0.01, ***P<0.001 compared with Sham; ^#^*P*<0.05, ^##^*P*<0.01 compared with I/R + NC.

To clarify the downstream mechanism of *miR-375*/CTGF-mediated protection from injury of cerebral I/R, we observed histological integrity and tissue damage in rat brains after subjecting to I/R. As indicated in Figure 3A, the results from H&E staining showed that profound degeneration and zonal cytoplasmic vacuolization were observed in I/R rat brain treated with NC, while injection with *miR-375* mimic clearly attenuated the degeneration and cytoplasmic vacuolization induced by I/R. Furthermore, a large number of TUNEL-positive cells was observed in the brain sections of rats subjected to I/R injury, whereas TUNEL-positive cells were significantly reduced in the *miR-375* mimic group compared with the NC group (Figure 3B), which was also reflected by the rate of quantitated apoptosis labeled by TUNEL staining (Figure 3D). Consistent with the observed improvement, Ctgf expression reflected by immunohistochemistry was decreased in *miR-375* mimic treated group (Figure 3C).

The ROS and MDA levels, known as the indication of intracellular oxidative stress, were significantly much higher in the I/R + NC group than in the Sham group. Of note, there was significant reduction in ROS and MDA level in the *miR-375* mimic-treated group compared with the NC-treated group (Figure 3E,F). In parallel with the decrease in ROS and MDA, we also observed that the activity of SOD, a key antioxidant enzyme, was decreased in I/R + NC group and significantly restored by *miR-375* mimic treatment (Figure 3G). Collectively, these findings indicate that *miR-375* could exert protective role against cerebral I/R injury through suppressing apoptosis and peroxidation.

### *MiR-375* promotes the proliferation and migration and inhibits the apoptosis in H/R PC12 cells

**Figure 4:**
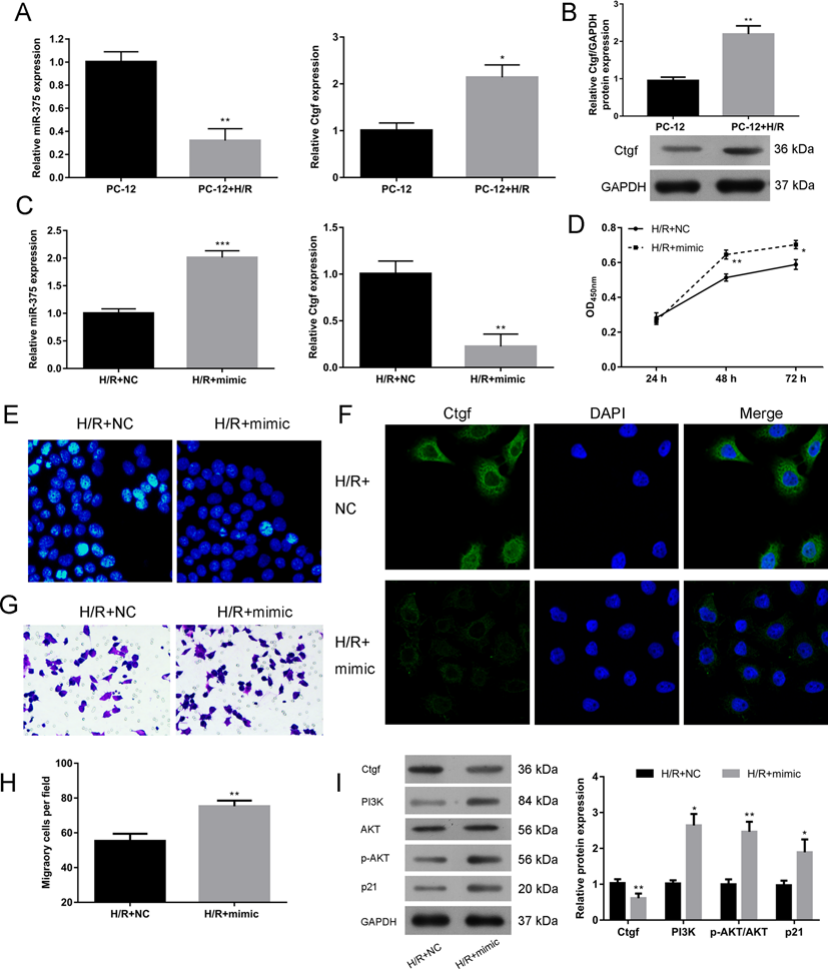
*MiR-375* promoted the proliferation and migration, and inhibited the apoptosis in H/R PC12 cells (**A**,**B**) The RNA expressions of *miR-375* and Ctgf (A) and the protein expression of Ctgf (B) in PC12 cells subjected to H/R or not. (**C**) The RNA expressions of *miR-375* and Ctgf in H/R PC12 cells treated with NC or *miR-375*. The expression of RNA was determined by qRT-PCR, and the expression of protein was determined by Western blotting. (**D**) The effect of *miR-375* on H/R p12 cells proliferation was detected by CCK-8 assay. (**E**) The effect of *miR-375* on H/R p12 cells apoptosis was examined by Hoechst 33258 staining. (**F**) The expressions of Ctgf in H/R PC12 cells treated with NC or *miR-375* was detected by immunofluorescence staining. (**G**,**H**) The effect of *miR-375* on H/R p12 cell migration was detected by transwell assay, and the migrant cells were calculated. (**I**) The effect of *miR-375* on protein expression of Ctgf, p21, PI3K, and p-AKT in H/R p12 cells was examined by Western blotting. The experiments were performed in triplicate and each value represents mean ± S.D. ***P<0.001, **P<0.01, *P<0.05.

To further prove the mechanism of *miR-375* underlying its protection from cellular H/R injury, H/R P12 cell model was established through 6 h of hypoxia and 48 h of reoxygenation. In line with the *in vivo* results, the expression of *miR-375* was decreased and mRNA and protein expression of Ctgf was elevated in H/R P12 cells (Figure 4A,B). After treating H/R P12 cells with NC or miR-375 mimic, we found that miR-375 mimic significantly suppressed mRNA expression of Ctgf (Figure 4C).

To determine the effects of *miR-375* levels on H/R P12 cell growth, cell viability was assessed through CCK-8 assay. H/R PC12 cells treated with *miR-375* mimic showed increased cell viability compared with H/R P12 cells with NC both 48 and 72 h after H/R (Figure 4D). In addition, the cell apoptosis indicated by Hoechst 33258 staining was much lower in the *miR-375* mimic-treated H/R PC12 cells than in the NC-treated group (Figure 4E).

Considering that cell migration plays an important role in the injury-induced neurogenesis and tissue regeneration [13,14]_._ For example, progenitor cells from periventricular region proliferate and migrate into the hippocampus to regenerate new neurones after ischemia and reduce neurological deficits *in vivo*. Herein, we thus explored effects of *miR-375* on PC12 cell migration. The results showed that *miR-375* mimic significantly promoted the migration of H/R PC12 cells, as indicated by transwell assay (Figure 4G,H). In the meantime, we found that the expression of Ctgf was decreased after treatment with *miR-375* mimic (Figure 4F).

It has been known that the PI3K/AKT signaling and p21 are closely related to cell survival, growth, and migration [15,16]. Thus, we next determined whether this protective role of *miR-375* against H/R injury was associated with PI3K/AKT and p21-dependent signaling pathway. As expected, *miR-375* mimic enhanced the protein expression of PI3K, p-AKT as well as p21 in H/R PC12 cells (Figure 4I).

Taken together, these results indicate that *miR-375* inhibited the expression of Ctgf in PC12 cells and protected the cells from H/R-induced injury through promoting cell proliferation and migration by p21/PI3K/AKT signaling pathways.

### *MiR-375* attentuates the H/R injury at least partly by silencing the expression of Ctgf

**Figure 5:**
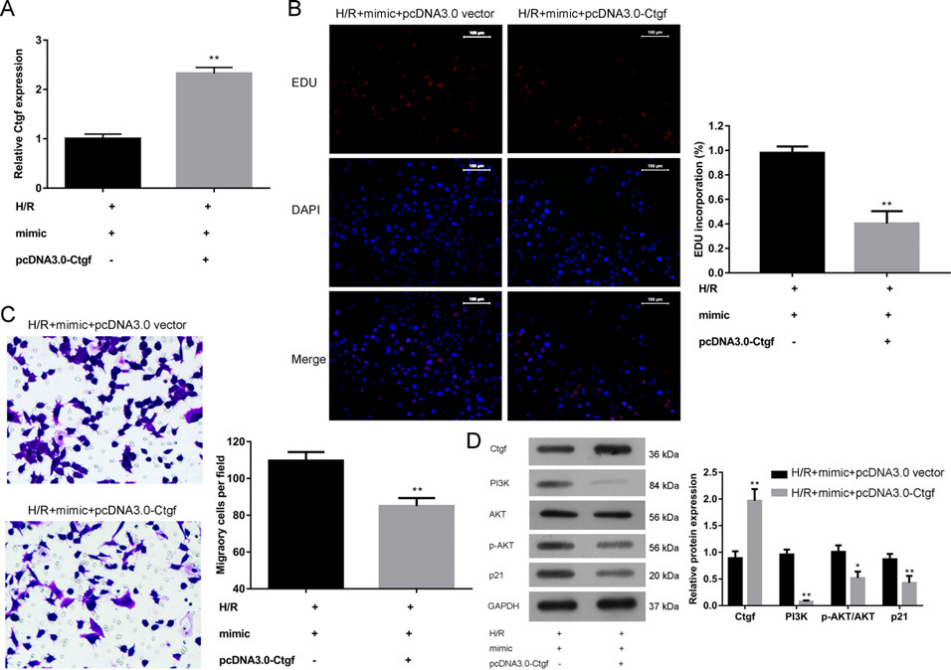
Ctfg suppressed the proliferation and migration in H/R PC12 cells (**A**) The mRNA expression of Ctgf in H/R PC12 cells transfected with *miR-375* mimic and pcDNA3.0-Ctgf or pcDNA3.0-vector was determined by qRT-PCR (**B**) The effect of *miR-375* mimic and pcDNA3.0-Ctgf co-transfection on H/R p12 cells proliferation was detected by EdU assay, with pcDNA3.0-vector as control. (**C**) The effect of *miR-375* mimic and pcDNA3.0-Ctgf co-transfection on H/R PC12 cells’ migration was detected by transwell assay. (**D**) The effect of *miR-375* mimic and pcDNA3.0-Ctgf co-transfection on protein expression of Ctgf, p21, PI3K, and p-AKT in H/R PC12 cells were determined by Western blotting. The experiments were performed in triplicate and each value represents mean ± S.D. *P<0.05 compared with H/R + mimic + pcDNA3.0 vector. **P<0.01 compared with H/R + mimic + pcDNA3.0 vector.

As shown in the above studies, Ctgf is a direct target of *miR-375*. We thus hypothesized that *miR-375* attenuated the H/R injury through inhibiting the expression of Ctgf. To confirm this, we co-transfected *miR-375* mimic and pcDNA3.0-Ctgf or pcDNA3.0 vector to verify the effect of Ctgf on H/R PC12 cell proliferation and migration. As indicated in Figure 5A, inhibited expression of *Ctgf* mRNA in *miR-375* mimic-transfected group was obviously restored by the addition of pcDNA3.0-Ctgf. Consistent with restored Ctgf expression in the presence of pcDNA3.0-Ctgf, H/R PC12 cell proliferation reflected by EdU assay was decreased in the group treated with pcDNA3.0-Ctgf compared with the group without pcDNA3.0-Ctgf (Figure 5B). Similarly, migration of H/R PC12 cell treated with pcDNA3.0-Ctgf was also reduced compared with the group without pcDNA3.0-Ctgf (Figure 5C). Moreover, PI3K, p-AKT and p21 expressions in the downstream of Ctgf were inhibited by pcDNA3.0-Ctgf (Figure 5D). These findings suggest that miRNA-375-mediated proliferation and migration of H/R PC12 cells is at least partly linked to inhibition of Ctgf expression.

## Discussion

Previous studies have demonstrated that expressions of miRNAs would be changed in the brain in response to I/R [9]. Elucidation of specific miRNA is thus considered as a potential target against I/R injury. In the present study, we demonstrated that *miR-375* expression is significantly down-regulated in the rat brain with I/R. With the *in vivo* and *in vitro* I/R stroke models, we found that *miR-375* mimic provides significant protection from injury of cerebral I/R, which is reflected by reduced infarct volumes and cell apoptosis, increased proliferation and migration of PC12 cells. Mechanistically, our findings showed that *miR-375* binds to 3′-UTR region of *Ctgf* mRNA, subsequently leading to the decreased expression of Ctgf in the I/R brain. Furthermore, we showed that *miR-375*/Ctgf-mediated protective effects are associated with p21/PI3K/Akt signaling pathways.

*miR-375* was originally identified from murine β-cells as a regulator of insulin secretion [17]. At present, *miR-375* has been shown to be found in multiple tissues or organs including brain [18–20]. Accumulating evidence on the comparison of miRNA expression profiles has demonstrated that *miR-375* expression was down-regulated in several neural injury models including cerebral I/R injury [9,10]. However, it is not clear that *miR-375* level in the brain was associated with the increased injury induced by I/R. In agreement with previous study [9], *miR-375* expression was significantly decreased in the I/R rat brain in our study. Importantly, we for the first time confirmed that *miR-375* does play a positive role in the protection from cerebral I/R injury, which is shown by overexpression of *miR-375* in the *in vivo* and *in vitro* I/R models.

Target genes of *miR-375* have been reported to include 3′-phosphoinositide-dependent protein kinase-1, hipposignaling effector YAP, the *p53* gene and SP1, most of which are involved in the development and progression of tumor [21–24]. Herein, we elucidated a new target Ctgf of *miR-375* using bioinformatics software of MirTarget algorithm and miRDB and a Dual-luciferase reporter assay. Ctgf, also known as CCN2, was first identified in 1991 to describe a novel polypeptide growth factor secreted by human endothelial cells [25]. Ctgf has been known to play important roles in many biological processes, including cell adhesion, migration, proliferation, angiogenesis, skeletal development, and tissue wound repair [26]. Recently, several studies showed that Ctgf levels have positive correlations to cell apoptosis in some specific pathological conditions. For instance, Ctgf has been shown to perform the pro-apoptotic effects of TGF-β1 on ovarian low-grade serous carcinoma [26]. Ctgf has been found to enhance the pro-apoptotic activity of glial-derived TGF-β2 and decrease the survival of periglomerular inhibitory neurones [27]. Similarly, Ctgf accumulation in the brain was increased in the rat traumatic brain injury [12]. In line with this, Ctgf expression was significantly increased in the rat I/R brain compared with non-I/R brain. Moreover, we showed that overexpression of *miR-375* leads to Ctgf down-regulation, which subsequently markedly reduced the injury induced by I/R, which was reflected by increased proliferation, migration, and decreased apoptosis of PC12 cells. Meanwhile, overexpression of Ctgf suppressed *miR-375*-mediated proliferation and migration in the H/R PC12 cells. Combined with other results, our findings suggest that Ctgf is one of the pivotal factors contributing to the injury of cerebral I/R. Also, *miR-375*-mediated protection from cerebral I/R injury was at least in part linked to down-regulation of Ctgf expression.

In summary, we here identified *miR-375* as a key factor which provides the protection from cerebral I/R injury. Inhibition of Ctgf, a target gene of *miR-375*, is an important reason of *miR-375*-mediated protective effects. Thus, our study suggests that Ctgf should be a potential therapeutic target against the injury of cerebral I/R.

## Abbreviations

CCK-8: cell counting kit-8
Ctgf: connective tissue growth factor
FNS: fastigial nucleus electrostimulation
H&E: Hematoxylin and Eosin
H/R: hypoxia/reoxygenation
I/R: ischemia/reperfusion
MCAO: middle cerebral artery occlusion
MDA: malondialdehyde
NC: negative control
ROS: reactive oxygen species
TTC: 2,3,5-Triphenyltetrazolium Chloride
TUNEL: terminal deoxynucleotidyl transferase mediated dUTP nick-end labeling
